# Efficacy and Safety of Remimazolam Compared to Midazolam for Sedation During Endoscopic Ultrasonography: A Single‐Center Retrospective Cohort Study

**DOI:** 10.1002/deo2.70267

**Published:** 2026-02-04

**Authors:** Haruka Toyonaga, Makoto Masaki, Hajime Yamazaki, Arata Oka, Yoshiki Matsuno, Hidetoshi Nakata, Shoji Takayama, Tatsuya Nakagawa, Takuya Takayama, Masahiro Orino, Hironao Matsumoto, Kimi Sumimoto, Masahiro Takeo, Norimasa Fukata, Takeshi Yamashina, Masaaki Shimatani, Makoto Naganuma

**Affiliations:** ^1^ Department of Gastroenterology and Hepatology Kansai Medical University Medical Center Osaka Japan; ^2^ Department of Community Medicine Section of Clinical Epidemiology Graduate School of Medicine Kyoto University Kyoto Japan; ^3^ Third Department of Internal Medicine Division of Gastroenterology and Hepatology Kansai Medical University Hospital Osaka Japan

**Keywords:** endoscopic ultrasonography, midazolam, pancreatobiliary diseases, remimazolam, sedation

## Abstract

**Objectives:**

Remimazolam is a novel ultra–short‐acting benzodiazepine that may offer advantages over conventional sedatives in endoscopic procedures. Evidence for its use in pancreatobiliary endoscopic ultrasonography (EUS) is limited. We compared the efficacy and safety of remimazolam and midazolam in outpatient pancreatobiliary EUS.

**Methods:**

This retrospective study included outpatients undergoing diagnostic pancreatobiliary EUS between July 2024 and July 2025. Patients received either remimazolam (initial 0.2 mg/kg, top‐up 0.1 mg/kg [high‐risk patients: 0.16 mg/kg, 0.08 mg/kg]) or midazolam. The target sedation depth was Modified Observer's Assessment of Alertness/Sedation ≤3, procedures were performed without supplemental oxygen, and pentazocine was co‐administered. The primary outcome was rapid recovery (meeting all criteria: modified Aldrete score ≥6 at 5 min, ≥9 at 30 min, ambulation ≥3 m at 30 min). Secondary outcomes included sedation success (all: EUS completion, without >2 top‐ups within 10 min, without rescue sedation, without agitation requiring interruption), recovery time, flumazenil use, and adverse events.

**Results:**

We analyzed 139 patients (remimazolam, *n* = 75; midazolam, *n* = 64). Rapid recovery was more frequent with remimazolam (70.7% vs. 25.0%, *p* < 0.001). Sedation success was higher (92.0% vs. 73.4%, *p* = 0.005), recovery was shorter (median 34 vs. 55 min, *p* < 0.001), and flumazenil use was lower (4.0% vs. 45.3%, *p* < 0.001). Hypoxemia (SpO_2_ <90% lasting ≥10 s) occurred slightly more often with remimazolam (30.6% vs. 26.6%, *p* = 0.707), but all episodes were mild and reversible with airway support/oxygen. Hypotension was rare and comparable.

**Conclusions:**

Remimazolam provided faster recovery and higher sedation success than midazolam in outpatient pancreatobiliary EUS. Supplemental oxygen before sedation is a reasonable option to enhance safety.

## 1 Introduction

Endoscopic ultrasonography (EUS) is pivotal in the diagnosis and management of pancreatobiliary diseases, enabling high‐resolution imaging, tissue acquisition, and transmural drainage [1−4]. Compared with esophagogastroduodenoscopy (EGD), pancreatobiliary EUS requires insertion of a large‐diameter echoendoscope, longer and more intense manipulation, and stricter control of patient movement. Consequently, adequate and stable sedation is essential−particularly in outpatients, where rapid induction and recovery enable efficient workflow and safe discharge. Given these requirements, findings from randomized trials in EGD cannot be directly generalized to EUS.

Midazolam, propofol, and dexmedetomidine are widely used, but each has notable limitations. Midazolam has long been standard for endoscopic sedation owing to its relatively rapid onset and reversibility with flumazenil; however, unpredictable pharmacokinetics, delayed recovery, and residual drowsiness remain important limitations in outpatient practice [5−7]. Propofol provides a rapid onset and recovery [8], yet carries a risk of respiratory depression and lacks an antagonist [9−11]. Dexmedetomidine preserves respiratory drive but has a slow onset and hemodynamic effects [12−14]. These limitations highlight the need for sedative regimens that provide stable sedation while facilitating rapid recovery in routine EUS.

Remimazolam besylate (Anerem, Mundipharma Co., Japan) is an ultra–short‐acting benzodiazepine metabolized by tissue esterases and reversible with flumazenil [15, 16]. Several randomized trials in EGD have demonstrated that remimazolam provides effective sedation comparable to midazolam, with faster recovery and favorable safety profiles; however, evidence in pancreatobiliary EUS outpatients is limited. Therefore, we aimed to compare the efficacy and safety of remimazolam and midazolam for sedation in outpatients undergoing pancreatobiliary EUS, with particular focus on sedation success and rapid recovery.

## 2 Methods

### 2.1 Study Design and Patients

We retrospectively analyzed consecutive outpatients undergoing diagnostic pancreatobiliary EUS with remimazolam or midazolam between July 2024 and July 2025 at Kansai Medical University Medical Center.

#### 2.1.1 Eligibility Criteria

Inclusion: Adults (≥18 years) undergoing outpatient pancreatobiliary EUS under sedation.

Exclusion: Missing key sedation/monitoring data; pregnancy or lactation; recent investigational drug use; inability to ambulate; severe baseline cardiorespiratory instability.

### 2.2 Sedation Procedure

Target depth was Modified Observer's Assessment of Alertness/Sedation ≤3 (Table 1) [17]. All patients received topical lidocaine (40 mg), pentazocine, and antispasmodics (scopolamine butylbromide or glucagon), with additional doses as needed. Standard monitoring included continuous electrocardiogram (ECG), heart rate, and SpO_2_, with automated noninvasive blood pressure every 5 min; oxygen was not administered routinely. Owing to intermittent supply, remimazolam was mainly used in July–October 2024 and February–July 2025; otherwise, midazolam was used. Dosing followed published regimens for EGD/ERCP [18−21], with risk‐based adjustments: **Remimazolam** 0.2 mg/kg initial bolus (capped at 15 mg) with 0.1 mg/kg top‐ups; high‐risk patients (age ≥75 years or American Society of Anesthesiologists‐Physical Status [ASA‐PS] ≥3) received 80% of these doses. **Midazolam** 2–4 mg initial bolus (1–3 mg in high‐risk patients) with 1–3 mg top‐ups at the physician's discretion.

**TABLE 1:** Description of Modified Observer's Assessment of Alertness/Sedation scores (MOAA/S) ^17^

Score	Description
**5**	Responds readily to a name spoken in a normal tone
**4**	Lethargic response to a name spoken in a normal tone
**3**	Responds only after the name is called loudly and/or repeatedly
**2**	Responds only after mild prodding or shaking
**1**	Responds only after a painful trapezius squeeze
**0**	No response after painful trapezius squeeze

### 2.3 Sedation Assessment

When remimazolam was first introduced, a standardized “procedure and sedation record sheet” was developed for all cases to optimize dosing, monitor safety, and document sedation‐related events. This sheet captured sedation‐ and procedure‐related parameters, including patient background and risk factors, sedative type and dose, timing of additional doses, vital signs, oxygen use, intra‐procedural events, and recovery status. The sheet was completed jointly by the endoscopists and nursing staff during each examination. Sedation‐related behaviors—such as agitation requiring interruption, arousal or body movement during scope insertion, and adverse events —were documented using predefined shared definitions after discussion between physicians and nursing staff. Variables were extracted according to the outcome definitions below.

### 2.4 Definition of Sedation Success

**Sedation success** was defined as 1) completion of the procedure, 2) without requiring top‐up sedatives more than twice within any 10‐min period, 3) without rescue sedatives (dexmedetomidine, propofol, or other benzodiazepines), and 4) without agitation requiring interruption (vigorous movement with attempts to remove the endoscope).

### 2.5 Endoscopic Ultrasound Procedure

All EUS procedures were conducted by experts (≥5 years; ≥500 pancreatobiliary EUS), supervised trainees using GF‐UCT260 (Olympus Co., Tokyo, Japan) or EG‐580UT/EG‐740UT (Fujifilm Co., Tokyo, Japan).

### 2.6 Definition of Adverse Events

The definitions of each adverse event are as follows:

**Hypoxemia;** SpO_2_< 90% lasting 10 s or longer. Supplemental oxygen before sedation was not administered routinely. **Hypotension;** systolic BP < 80 mmHg or a decrease of ≧30% from baseline. **Hypertension;** systolic BP >180 mmHg or an increase of ≧30% from baseline. **Bradycardia;** <50 beats per minute or a decrease of ≧30% from baseline. **Injection pain;** painful sedative injection. **Post‐operative nausea and vomiting (PONV);** nausea and vomiting that occur after EUS procedures.

### 2.7 Recovery Assessment and Definition of Rapid Recovery

The level of consciousness was assessed using the modified Aldrete score (Table 2) [22] at 5 and 30 min after EUS procedures. Patients with a score ≥6 at 5 min were transferred from the examination room to the recovery area. Discharge required a score ≥9 and stable ambulation ≥3 m at 30 min post‐procedure.

**TABLE 2:** Description of Modified Aldrete score.[22]

Assessment items	Condition	Grade
**Activity**	Able to move all four extremities voluntarily or on command	2
	Able to move all two extremities voluntarily or on command	1
	Unable to move all four extremities voluntarily or on command	0
**Respiration**	Able to breathe deeply and cough freely	2
	Dyspnea or limited breathing	1
	Apneic	0
**Circulation**	Blood pressure within ±20% of pre‐anesthetic level	2
	Blood pressure within ±20%–49% of pre‐anesthetic level	1
	Blood pressure deviation ≥50% from pre‐anesthetic level	0
**Consciousness**	Fully awake	2
	Arousable on calling	1
	Not responding	0
**Oxygen saturation**	Able to maintain SpO_2_ ≥92% on room air	2
	Needs supplemental oxygen to maintain SpO_2_ ≥ 90%	1
	SpO_2_ < 90% even with supplemental oxygen	0

Rapid recovery required all three criteria: the transfer criterion at 5 min, the discharge criterion at 30 min, and stable ambulation ≥3 m at 30 min. Ambulation was additionally assessed to ensure sufficient recovery beyond the physiological criteria of the Aldrete score. Conversely, those who failed to meet any of these criteria were classified as having delayed recovery. In such cases, additional rest was provided, and flumazenil (0.5 mg) was administered.

### 2.8 Primary Outcome

The primary outcome was the proportion of patients with **rapid recovery** (composite definition above).

### 2.9 Secondary Outcomes

The secondary outcomes were as follows; (1) sedation success rate (as defined above) and its components; (2) induction time; (3) induction achievement with the initial dose; (4) endoscope insertion without arousal due to discomfort; (5) number of body movements during the procedure; (6) incidence of agitation requiring interruption; (7) incidence of adverse events; (8) management for hypoxemia; (9) modified Aldrete scores at 5 and 30 min; (10) recovery time (from scope withdrawal to meeting all discharge criteria); (11) usage rate of flumazenil; (12) Memory retention during the procedure.

### 2.10 Statistical Analysis

Continuous variables were presented as medians and ranges (Mann‐Whitney U test); categorical variables as proportions (Fisher's exact test). Standardized mean differences (SMDs) were calculated to assess the balance of baseline characteristics between groups. To evaluate the association of the sedative agent (remimazolam vs. midazolam) with rapid recovery and sedation success, multivariable logistic regression was performed to estimate adjusted odds ratios (aORs) with 95% confidence intervals using two models. Model 1 was a full model adjusted for age, sex, body‐mass index (BMI), ASA‐PS (I/II vs. III/IV), Mallampati class (I/II vs. III/IV), procedure period (Early: July 2024–Jan 2025; Late: Feb–Jul 2025), and history of alcohol consumption or chronic use of sedative/psychotropic medications. Model 2 was a reduced model adjusted for key confounders only (age, ASA‐PS, and history of alcohol consumption or chronic use of sedative/psychotropic medications). Both models were used to confirm the robustness of our findings. The full multivariable analysis results, including adjusted odds ratios for all covariates, are shown in Table S1. A p‐value of <0.05 was considered statistically significant. All statistical analyses were performed using JMP Student Edition version 18.2.0 (SAS Institute Inc., Cary, NC, USA).

## 3 Results

A total of 139 patients were included: 75 received remimazolam and 64 received midazolam. Baseline characteristics, including age, sex, ASA‐PS, Mallampati classification, and comorbidities (Table 3), were comparable between groups.

**TABLE 3:** Patients’ characteristics, grouped by sedative: Remimazolam or Midazolam.

	Remimazolam group (*n* = 75)	Midazolam group (*n* = 64)	*p*‐Value	SMD
Median age, years (range)	72.0 (44–88)	74.0 (18–88)	0.358	0.091
Sex, men, n(%)	36 (48.0)	27 (42.2)	0.50	0.117
Median body weight, kg (range)	55.0 (30.8–101.0)	55.0 (30.5–84.0)	0.886	0.019
Median BMI, kg/m^2^ (range)	21.80 (12.80–35.00)	22.10 (14.60–31.60)	0.758	0.055
ASA‐PS I/II/III/IV, *n* (%)	8/49/18/0	3/46/15/0	0.622	0.229
Mallampati classification I/II/III/IV, *n* (%)	35/37/1/2	30/30/4/0	0.319	0.328
Comorbidity, *n* (%)				
Cardiovascular disease	4 (5.3)	3 (4.7)	1.00	0.030
Respiratory disease	8 (10.7)	4 (6.2)	0.385	0.160
Cerebrovascular disease	4 (5.3)	3 (4.7)	1.00	0.030
Renal dysfunction	24 (32.0)	30 (46.9)	0.083	0.306
Hypertension	33 (44.0)	36 (56.2)	0.175	0.246
Diabetes	25 (33.3)	22 (34.4)	1.00	0.022
History of smoking, *n* (%)	33 (44.0)	27 (42.2)	0.865	0.037
Alcohol abuse, *n* (%)	15(20.0)	11 (17.2)	0.828	0.072
Regular use of hypnotics and psychotropic drugs, *n* (%)	10 (13.3)	11 (17.2)	0.636	0.107
Sedation risk, normal‐risk/high‐risk (%)	33 (44.0)/42 (56.0)	25 (39.1)/39 (60.9)	0.607	0.100
Indication for EUS, *n* (%)				
Pancreatic cysts	47 (62.7)	33 (51.6)	0.229	0.225
Pancreatic tumors	8 (10.7)	5 (7.8)	0.771	0.099
Biliary stones	3 (4.0)	3 (4.7)	1.00	0.034
Biliary tumors	2 (2.7)	3 (4.7)	0.661	0.320
Abnormal blood test results	3 (4.0)	5 (7.8)	0.470	0.164
Pancreatic dilation or stricture	1 (1.3)	2 (3.1)	0.595	0.124
Ampullary tumors	2 (2.7)	1 (1.6)	1.0	0.077
Pancreatitis	9 (12.0)	8 (12.5)	1.0	0.015
Others^*^	0 (0.0)	4 (6.2)	N/A	0.498
Endoscopist experience, *n* (%)			0.858	0.055
Experts	50 (66.7)	41 (64.1)		
Trainees	25 (33.3)	23 (35.9)		

For continuous data, the medians and ranges are shown. The Mann–Whitney U test and Fisher's exact test were used.

SMD, standardized mean difference; BMI, body mass index; ASA‐PS, American society of anesthesiologists physical status; N/A, not applicable

* Others: Lymphadenopathy 2, pancreaticobiliary maljunction 1, retroperitoneal tumor 1

### 3.1 Sedation Procedure

In the remimazolam group, the median initial dose was 10 mg (range, 5–15 mg), and the median top‐up dose was 5 (0–8) mg. In the midazolam group, the median initial dose was 3 (2–4) mg, and the median top‐up dose was 2 (0–3) mg. Doses by risk category are detailed in Table 4.

**TABLE 4:** Details of sedation procedure for endoscopic ultrasonography (EUS).

	Remimazolam group (*n* = 75)	Midazolam group (*n* = 64)	*p*‐Value
Risk group (normal‐risk/high‐risk), *n* (%)	33 (44.0)/42 (56.0)	25 (39.1)/39 (60.9)	0.607
Initial dose of sedative, mg (median: range)	10 (5–15)	3 (2–4)	N/A
for normal‐risk patients	12 (9–15)	3 (3–4)	N/A
for high‐risk patients	8 (5–14)	3 (2–3)	N/A
Top‐up dose of sedative, mg (median: range)	5 (0–8)	2 (0–3)	N/A
for normal‐risk patients	6 (0–8)	2 (1–3)	N/A
for high‐risk patients	4 (0–8)	2 (0–2.5)	N/A
Number of top‐up doses required for sedation induction, *n* (median: range)	0 (0–0)	0 (0–4)	<0.001
Number of top‐up doses during the procedure, *n* (median: range)	2 (0–4)	2 (0–5)	0.557
Total dose of sedative, mg (median: range)	16 (7–36)	6 (2–13)	N/A
for normal‐risk patients	20 (10–36)	7 (4–16)	N/A
for high‐risk patients	15 (7.5–30)	5 (2–13)	N/A
Pentazocine dose, *n* (%)			0.551
7.5mg	58 (77.3)	46 (71.9)	
15mg	16 (21.3)	16 (25.0)	
22.5mg	0 (0)	1 (1.6)	
Rate of haloperidol administration, *n* (%)	5 (6.7)	4 (6.3)	1
Total procedure time, minutes (median: range)	26 (7–47)	27 (10–47)	0.222

For continuous data, the medians and ranges are shown. The Mann–Whitney U test and Fisher's exact test were used.

In the remimazolam group, normal‐risk patients received 0.2 mg/kg initially and 0.1 mg/kg top‐ups, while high‐risk patients received 0.16 mg/kg initially and 0.08 mg/kg top‐ups. In the midazolam group, normal‐risk patients received an initial dose of 2–4 mg, with 2–3 mg top‐ups, while high‐risk patients received 1–3 mg initially and 1–2 mg top‐ups. Dosages were adjusted at the physician's discretion.

N/A: not applicable

Top‐up doses for induction were significantly lower with remimazolam (median 0 [0–0]) than in the midazolam group (0 [0–4], *p* < 0.001), while intraprocedural top‐ups were similar. Pentazocine dose and haloperidol administration rates were comparable between groups, as was total procedure time.

### 3.2 Primary Outcome

**FIGURE 1:**
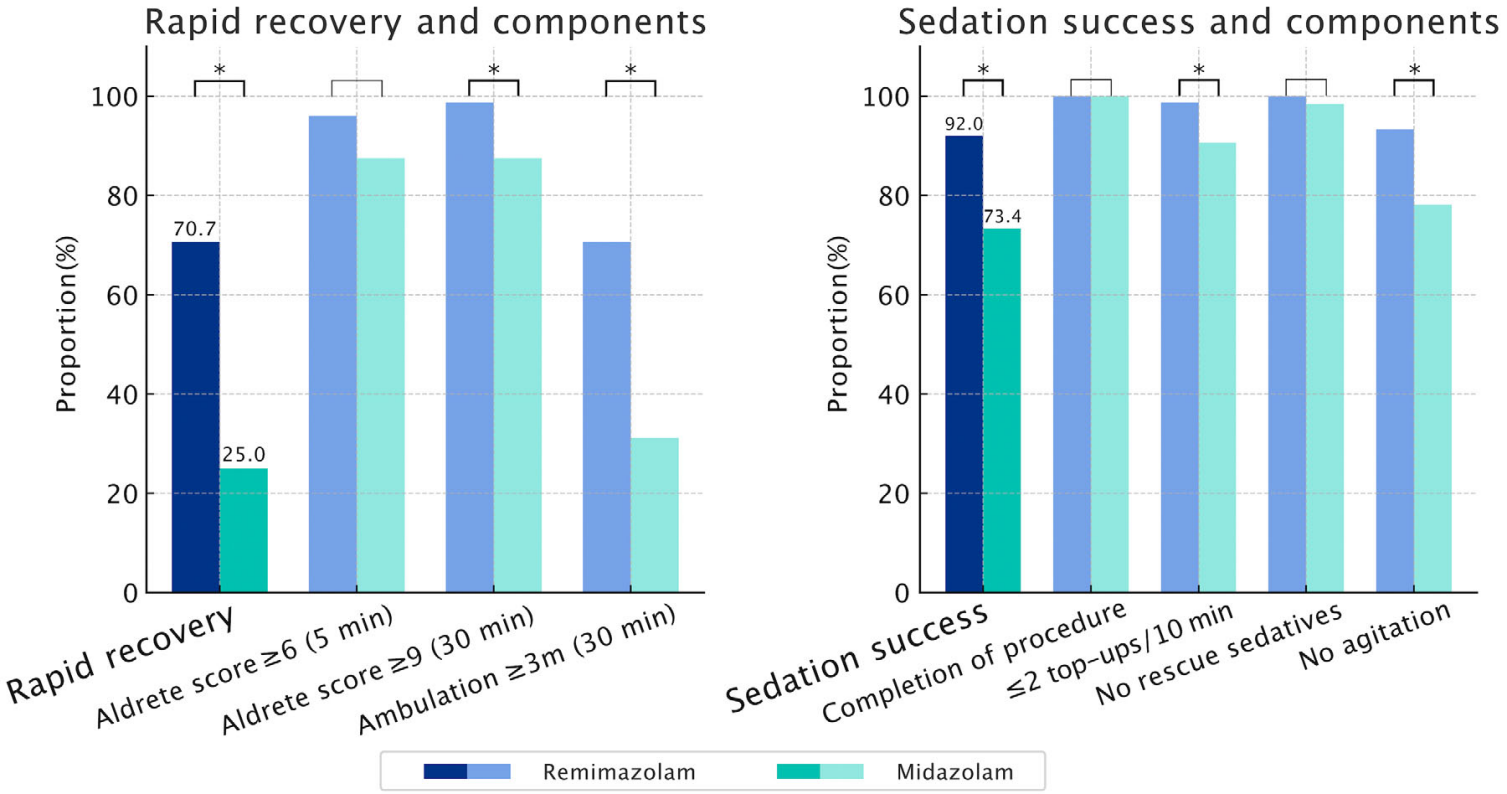
Outcomes of sedation during outpatient pancreatobiliary endoscopic ultrasonography (EUS). Rates of rapid recovery and sedation success are shown for remimazolam (blue) and midazolam (green). An asterisk (*) indicates a statistically significant difference between groups (*p* < 0.05).

As shown in Table 5 and Figure 1, the proportion of patients achieving rapid recovery was significantly higher in the remimazolam group than in the midazolam group (70.7% vs. 25.0%, *p* < 0.001). For the individual components of the composite outcome, a modified Aldrete score ≥6 at 5 min post‐procedure was achieved in 96.0% of patients in the remimazolam group and 87.5% in the midazolam group, with no statistically significant difference (*p* = 0.112). A modified Aldrete score ≥9 at 30 min was observed in 98.7% versus 87.5%, respectively, showing a significant difference (*p* = 0.012). Stable ambulation ≥3 m at 30 min was significantly more frequent in the remimazolam group (70.7%) compared with the midazolam group (31.2%, *p* < 0.001).

**TABLE 5:** Outcomes of sedation for endoscopic ultrasonography (EUS).

	Remimazolam group (*n* = 75)	Midazolam group (*n* = 64)	*p*‐Value
**Primary outcome**			
Rapid recovery, *n* (%)	53 (70.7)	16 (25.0)	<0.001
Aldrete score ≥6 at 5 min, *n* (%)	72 (96.0)	56 (87.5)	0.112
Aldrete score ≥9 at 30 min, *n* (%)	74 (98.7)	56 (87.5)	0.012
Stable ambulation ≥3 m at 30 min, *n* (%)	53 (70.7)	20 (31.2)	<0.001
**Secondary outcomes**			
Sedation success, *n* (%)	69 (92.0)	47 (73.4)	0.005
Completion of procedures, *n* (%)	75 (100)	64 (100)	1.00
No requirement for more than twice the top‐up dose within 10 min, *n* (%)	74 (98.7)	58 (90.6)	0.048
No requirement for rescue sedatives, *n* (%)	75 (100)	63 (98.4)	0.460
Absence of agitation requiring interruption, *n* (%)	70 (93.3)	50 (78.1)	0.013
Induction time, min (median: range)	1 (1‐2)	1 (1‐11)	<0.001
Achieving sedation induction with the initial dose, *n* (%)	73 (97.3)	49 (76.6)	<0.001
Absence of arousal or body movement due to discomfort during scope insertion, *n* (%)	58 (77.3)	29 (45.3)	<0.001
Number of body movements during the procedure, *n* (median: range)	1 (0‐11)	1 (0‐11)	0.113
Incidence of agitation requiring interruption, *n* (%)	5 (6.7)	14 (21.9)	0.013
Incidence of adverse events, *n* (%)			
Hypoxemia	23 (30.6)	17 (26.6)	0.707
Hypotension	1 (1.3)	2 (3.1)	0.595
Hypertension	1 (1.3)	0 (0.0)	1.00
Bradycardia	0 (0)	0 (0)	1.00
Injection pain	0 (0)	2 (3.1)	0.210
Post‐operative nausea and vomiting	2 (2.7)	8 (12.5)	0.044
Dizziness	2 (2.7)	7 (10.9)	0.080
Headache	2 (2.7)	3 (4.7)	0.661
Management for hypoxemia, *n* (%)			
Airway maintenance	23 (30.6)	12 (18.8)	0.120
Supplemental oxygen administration	19 (25.3)	8 (12.5)	0.084
Positive pressure ventilation	0 (0)	0 (0)	1.00
Modified Aldrete score at 5 min (median: range)	10 (4‐10)	9 (3–10)	<0.001
Modified Aldrete score at 30 min (median: range)	10 (7–10)	10 (5–10)	0.001
Recovery time, median (range), min	34 (9–95)	55 (12–203)	<0.001
Requiring flumazenil administration, *n* (%)	3 (4.0)	29 (45.3)	<0.001
Memory retention during the procedure, *n* (%)	17 (22.7)	18 (28.2)	0.551

For continuous data, the medians and ranges are shown. The Mann–Whitney U test and Fisher's exact test were used.

Rapid recovery is defined by a composite measure of the following: 1) Modified Aldrete score ≥6 at 5 min post‐procedure, 2) Modified Aldrete score ≥9 at 30 min post‐procedure, and 3) Stable ambulation ≥3 m at 30 min post‐procedure

Sedation success was defined by a composite measure of the following: 1) Completion of the EUS procedure, 2) No requirement for top‐up sedative administration more than twice within 10 min, 3) No requirement for a rescue sedative with dexmedetomidine, propofol, or other benzodiazepine, 4) Absence of agitation requiring interruption of the EUS examination.

In univariate logistic regression, remimazolam use was associated with higher odds of rapid recovery (OR = 7.23, 95% confidence interval [CI] 3.40–15.30, *p* < 0.001) (Table 6).

**TABLE 6:** Multivariable analysis for the association of sedative agents with rapid recovery and sedation success

	Rapid recovery	Univariate analysis		Multivariate analysis			
				Model 1^*^		Model 2^†^	
	**Proportion**	**Odds ratio** **(95%CI)**	***p*‐Value**	**Odds ratio** **(95%CI)**	***p*‐Value**	**Odds ratio** **(95%CI)**	***p*‐Value**
Midazolam group (*n* = 64)	25.0% (16/64)	1.00 (reference)		1.00 (reference)		1.00 (reference)	
Remimazolam group (*n* = 75)	70.7% (53/75)	7.23 (3.40−15.30)	<0.001	7.93 (3.51−17.90)	<0.001	8.26 (3.73−18.30)	<0.001
	**Sedation success**	**Univariate analysis**		**Multivariate analysis**			
				**Model 1** ^*^		**Model 2** ^†^	
	**Proportion**	**Odds ratio** **(95%CI)**	***p*‐Value**	**Odds ratio** **(95%CI)**	***p*‐Value**	**Odds ratio** **(95%CI)**	***p*‐Value**
Midazolam group (*n* = 64)	73.4% (47/64)	1.00 (reference)		1.00 (reference)		1.00 (reference)	
Remimazolam group (*n* = 75)	92.0% (69/75)	4.16 (1.53−11.30)	0.005	4.75 (1.62−13.90)	0.005	4.63 (1.65−13.00)	0.004

The multivariable logistic regression analysis was performed using two models.

* Model 1 was a full model adjusted for age, sex, BMI, ASA‐PS (I/II vs. III/IV), Mallampati class (I/II vs. III/IV), procedure period (Early: July 2024–Jan 2025; Late: Feb–Jul 2025), and history of alcohol consumption or chronic use of sedative/psychotropic medications.

^†^ Model 2 was a reduced model adjusted for key confounders only (age, ASA‐PS, and history of alcohol consumption or chronic use of sedative/psychotropic medications).

In multivariable logistic regression, remimazolam remained strongly associated with rapid recovery in both Model 1 (aOR = 7.93, 95% CI 3.51–17.90, *p* < 0.001) and Model 2 (aOR = 8.26, 95% CI 3.73–18.30, *p* < 0.001), confirming the robustness of the findings. The full model results, including adjusted odds ratios for all covariates, are provided in Table S1.

### 3.3 Secondary Outcomes

Sedation success was significantly higher with remimazolam (92.0% vs. 73.4%, *p* = 0.005) (Table 5 and Figure 1). Univariate analysis demonstrated that remimazolam was associated with greater odds of sedation success (OR = 4.16, 95% CI 1.53–11.30, *p* = 0.005). In the multivariable analysis, this association remained independent in both Model 1 (aOR = 4.75, 95% CI 1.62–13.90, *p* = 0.005) and Model 2 (aOR = 4.63, 95% CI 1.65–13.00, *p* = 0.004).

The remimazolam group more frequently achieved sedation induction with the initial dose (97.3% vs. 76.6%, *p* < 0.001) and exhibited fewer cases of agitation requiring interruption (6.7% vs. 21.9%, *p* = 0.013). Absence of arousal or body movement during scope insertion was more common with remimazolam (77.3% vs. 45.3%, *p* < 0.001), although the total number of body movements during the procedure did not differ significantly.

Median induction time was 1 min in both groups, but the range was narrower for remimazolam (1–2 min vs. 1–11 min, *p* < 0.001).

The incidence of hypoxemia, hypotension, hypertension, bradycardia, injection pain, dizziness, and headache did not differ significantly between groups. Although the difference was not statistically significant, the incidence of hypoxemia (SpO_2_ < 90% lasting ≥10 s) was slightly higher in the remimazolam group than in the midazolam group (30.6% vs. 26.6%, *p* = 0.707). All episodes were mild, requiring only airway repositioning and/or supplemental oxygen. No cases necessitated procedure interruption, positive pressure ventilation, or endotracheal intubation. PONV was less frequent in the remimazolam group (2.7% vs. 12.5%, *p* = 0.044).

The median modified Aldrete score was higher in the remimazolam group than in the midazolam group at both 5 min (10 [4–10] vs. 9 [3–10], *p* < 0.001) and 30 min (10 [7–10] vs. 10 [5–10], *p* = 0.001). The recovery time was markedly shorter in the remimazolam group than in the midazolam group (34 min [9–95] vs. 55 min [12–203], *p* < 0.001). Flumazenil use was significantly lower in the remimazolam group (4.0% vs. 45.3%, *p* < 0.001). Memory retention rates during the procedure were similar.

## 4 Discussion

This single‐center retrospective cohort study suggests that remimazolam provides clinically meaningful advantages over midazolam for outpatient pancreatobiliary EUS. Remimazolam was associated with higher rates of rapid recovery and sedation success, while safety profiles were comparable. Flumazenil use was markedly lower, and PONV was less frequent with remimazolam. These findings support remimazolam as a pragmatic sedative option in outpatient EUS. Oxygen was not routinely administered, and consequently, some patients in both groups experienced hypoxemia (SpO_2_ < 90% lasting ≥ 10 s). However, all episodes were transient and mild, manageable with airway support or supplemental oxygen. For safer practice, routine supplemental oxygen before sedation is a reasonable consideration.

Several observations help explain these results. First, the number of top‐up doses required to achieve sedation induction was lower with remimazolam, and the range of induction time was narrower, indicating a more predictable onset. Second, agitation requiring interruption occurred less often, and scope insertion without arousal or body movement was more frequent in the remimazolam group, suggesting more stable sedation during the initial, stimulus‐intense phases of EUS. Together, these features likely contributed to the higher sedation success rate and the faster recovery we observed.

These findings align with remimazolam's pharmacologic profile: rapid esterase metabolism enables quick titration and recovery, and reliable flumazenil reversal reduces re‐sedation risk. Prior studies in upper and lower endoscopy have reported faster recovery with remimazolam, and our data extend these findings to pancreatobiliary EUS, where evidence has been limited. Beyond upper and lower endoscopy, a multicenter randomized controlled trial [23] in diagnostic EUS showed that remimazolam reduced cardiopulmonary adverse events compared with propofol, with faster induction and comparable recovery under routine oxygen supplementation.

Clinically, faster and more consistent recovery benefits outpatient EUS by improving throughput and enabling timely discharge. The combination of high sedation success and rapid recovery without an increase in serious adverse events suggests that remimazolam can streamline workflow while preserving safety. Notably, the benefit persisted after adjustment for age, sex, BMI, ASA‐PS, Mallampati class, procedure period, and alcohol/medication history, and the “procedure period” covariate itself was not independently associated with outcomes—mitigating concerns that temporal factors related to drug availability drove the findings.

At the time of study initiation, remimazolam had not yet been approved for endoscopic sedation in Japan. After our study was initiated, a Japanese multicenter phase III single‐arm study [24] reported high overall sedation success using a fixed‐dose regimen (3 mg induction with 1 mg top‐ups) across endoscopic procedures; however, hepatobiliary/pancreatic cases were very limited (*n* = 7), constraining inference for EUS. Therefore, our institutional dosing regimen was determined with reference to previous overseas protocols for gastrointestinal endoscopy and ERCP with adjustments for patient risk categories.

Previous studies have shown that remimazolam at 0.1–0.2 mg/kg is effective for gastrointestinal endoscopy [18, 25], while approximately 0.3 mg/kg is appropriate for ERCP, with lower incidences of cardiorespiratory events compared with midazolam or propofol [19, 21]. In addition to weight‐based regimens, several fixed‐dose strategies have been reported for EGD or colonoscopy, typically using 3–8 mg boluses with 1–3 mg top‐ups, and have demonstrated non‐inferiority to midazolam or propofol [26−28]. However, the Japanese EGD‐approved protocol (3 mg bolus with 1 mg top‐ups [29, 30]) often proved insufficient in our initial EUS experience, frequently requiring multiple additional doses and failing to maintain adequate sedation. These findings suggest that pancreatobiliary EUS, which requires deeper and more stable sedation, may be better managed with higher or weight‐based dosing strategies.

Although the incidence of hypoxemia was slightly higher in the remimazolam group than in the midazolam group, all episodes were mild, manageable with basic airway maneuvers or supplemental oxygen, and required no advanced interventions. Previous reports have also described transient, reversible hypoxemia with remimazolam [15, 25, 31, 32]. Notably, studies conducted without supplemental oxygen showed rates comparable to our findings [18], whereas those with oxygen supplementation reported much lower rates, suggesting that administering oxygen prior to sedation induction may be effective in reducing hypoxemia [20, 21, 25, 33]. The lower incidence of PONV in the remimazolam group may reflect a pharmacologic advantage, but given the frequent use of pentazocine, the influence of opioid‐related effects cannot be excluded and warrants cautious interpretation.

Remimazolam is more expensive than midazolam (JPY 2218 per 50‐mg vial vs. JPY 92–115 per 10‐mg ampoule), but frequent flumazenil (JPY 1005–2001 per 0.5 mg ampoule) use with midazolam makes the overall per‐case cost roughly comparable. In our cohort, shorter recovery and markedly less flumazenil use with remimazolam may further offset cost differences by improving turnover. Although the current 50‐mg vial leads to drug wastage for typical doses (∼16 mg), the availability of smaller vials could enhance cost‐effectiveness. Overall, remimazolam appears cost‐acceptable for outpatient EUS.

This study has limitations. It was retrospective and single‐center, with drug selection partly determined by supply availability, raising the possibility of residual confounding. Oxygen supplementation was not routinely administered, which may have contributed to the relatively high incidence of mild hypoxemia. A comprehensive cost‐effectiveness analysis was not performed and warrants future investigation. Finally, the sample size was limited, and larger multicenter studies are required to validate our findings.

Prospective multicenter trials are warranted to establish optimal dosing strategies and confirm these findings, particularly in elderly and high‐risk patients. In summary, remimazolam achieved faster recovery and higher sedation success than midazolam without increasing serious adverse events, supporting its potential as a safe and efficient sedative for outpatient pancreatobiliary EUS.

## Author Contributions

**Conception and design**: Haruka Toyonaga, Makoto Masaki, and Masaaki Shimatani. **Performed endoscopic procedures and managed patients**: Haruka Toyonaga, Makoto Masaki, Arata Oka, Yoshiki Matsuno, Hidetoshi Nakata, Shoji Takayama, Tatsuya Nakagawa, Takuya Takayama, KO, Hironao Matsumoto, Masahiro Takeo, Takeshi Yamashina, and Masaaki Shimatani. **Analysis and interpretation of the data**: Haruka Toyonaga, Makoto Masaki, and Hajime Yamazaki. **Drafting of the article**: Haruka Toyonaga. **Critical revision of the manuscript for important intellectual content**: All of the authors. **Final approval of the manuscript**: All of the authors.

## Conflicts of Interest

HY reports lecture fees from Janssen Pharmaceutical KK, Mitsubishi Tanabe Pharma, Kowa Co. Ltd, AstraZeneca KK, Kyorin Pharmaceutical Co. Ltd., Takeda Pharmaceutical Co. Ltd., and Mundipharma KK. Outside the submitted work, under contracts with Kyoto University, fees for consultation to HY were paid to Kyoto University by Takeda Pharmaceutical Co., Ltd. and Magmitt Pharmaceutical Co., Ltd.

## Funding

None

## Ethics Statement

Approval of the research protocol: The institutional review board approved clinical use of remimazolam for EUS (KMUMC IRB 2024‐1T, May 24, 2024), and the retrospective study was subsequently approved (IRB 2025181, Aug 8, 2025).

## Consent

A retrospective study was obtained with opt‐out consent via the hospital website. Clinical use of remimazolam for EUS, with written and verbal consent, and midazolam was used as a conventional sedative agent, with written informed consent obtained from all patients.

## Clinical Trial Registration

N/A

## Supporting information




**TABLE S1** Full results of multivariable logistic regression analysis for rapid recovery and sedation success.
