# Supplementary material for: Efficacy and Safety of Remimazolam Compared to Midazolam for Sedation During Endoscopic Ultrasonography: A Single‐Center Retrospective Cohort Study
Source: DEN Open. 2026 Feb 4;6(1):e70267. doi: 10.1002/deo2.70267 (PMC12872400; doi:10.1002/deo2.70267)
Supplement: Supplementary file 1 — TABLE S1 Full results of multivariable logistic regression analysis for rapid recovery and sedation success. [file DEO2-6-e70267-s001.docx]

| **Rapid recovery** |  | | | |  | | | |  | |
| --- | --- | --- | --- | --- | --- | --- | --- | --- | --- | --- |
| **Variable^‡^** | **Univariate analysis** | | | **Multivariate analysis** | | | | | | |
|  |  |  |  | **Model 1^*^** | | | | | **Model 2^†^** | |
|  | Odds ratio  (95%CI) | *p*-value | | Odds ratio  (95%CI) | | *p*-value | | | Odds ratio  (95%CI) | *p*-value |
| Sedative group (Remimazolam) | 7.23  (3.40−15.30) | <0.001 | | 7.93  (3.51−17.90) | | <0.001 | | | 8.26  (3.73−18.30) | <0.001 |
| Age  (per year) | 1.02  (0.98−1.05) | 0.275 | | 1.03  (0.99−1.08) | | 0.152 | | | 1.03  (1.00−1.07) | 0.089 |
| Sex  (Woman) | 0.65  (0.33−1.27) | 0.205 | | 0.59  (0.26−1.3) | | 0.199 | | |  |  |
| ASA-PS  (III/IV) | 0.68  (0.31−1.50) | 0.344 | | 0.53  (0.21−1.40) | | 0.201 | | | 0.55  (0.22−1.37) | 0.199 |
| BMI  (per kg/m^2^) | 0.93  (0.85−1.01) | 0.096 | | 0.94  (0.85−1.04) | | 0.232 | | |  |  |
| Mallampati classification  (III/IV) | 0.39  (0.07−2.07) | 0.268 | | 0.38  (0.05−2.66) | | 0.328 | | |  |  |
| Alcohol or sedative/psychotropic use (Present) | 1.09  (0.54−2.22) | 0.810 | | 0.87  (0.37−2.05) | | 0.755 | | | 1.08  (0.48−2.43) | 0.847 |
| Procedure period (Late) | 0.45  (0.22−0.92) | 0.029 | | 0.50  (0.22−1.16) | | 0.108 | | |  |  |
|  |  | |  |  | | |  | |  | |
| **Sedation success** |  | |  |  | | |  | |  | |
| **Variable^‡^** | **Univariate analysis** | | | **Multivariate analysis** | | | | | | |
|  |  |  |  | **Model 1^*^** | | | | **Model 2^†^** | | |
|  | Odds ratio  (95%CI) | *p*-value | | Odds ratio  (95%CI) | | *p*-value | | | Odds ratio  (95%CI) | *p*-value |
| Sedative group (Remimazolam) | 4.16  (1.53−11.30) | 0.005 | | 4.75  (1.62−13.90) | | 0.005 | | | 4.63  (1.65−13.00) | 0.004 |
| Age  (per year) | 1.03  (0.99−1.07) | 0.158 | | 1.04  (1.00−1.09) | | 0.065 | | | 1.03  (0.99−1.07) | 0.104 |
| Sex  (Woman) | 0.37  (0.13−0.99) | 0.048 | | 0.30  (0.10−0.91) | | 0.034 | | |  |  |
| ASA-PS  (III/IV) | 2.33  (0.65−8.39) | 0.197 | | 2.22  (0.56−8.84) | | 0.256 | | | 2.58  (0.67−9.99) | 0.169 |
| BMI  (per kg/m^2^) | 0.94  (0.85−1.05) | 0.264 | | 0.98  (0.85−1.12) | | 0.735 | | |  |  |
| Mallampati classification  (III/IV) | 0.47  (0.09−2.60) | N/A | | 0.46  (0.07−3.12) | | 0.430 | | |  |  |
| Alcohol or sedative/psychotropic use (Present) | 0.88  (0.34−2.25) | 0.787 | | 0.62  (0.21−1.80) | | 0.379 | | | 0.82  (0.30−2.22) | 0.694 |
| Procedure period (Late) | 1.01  (0.40−2.59) | 0.978 | | 1.39  (0.47−4.06) | | 0.551 | | |  |  |

**Supplementary Table S1.** Full results of multivariable logistic regression analysis for rapid recovery and sedation success.

ASA-PS, American society of anesthesiologists physical status; BMI, body mass index; N/A, not applicable

The multivariable logistic regression analysis was performed using two models.

*Model 1 was a full model adjusted for age, sex, BMI, ASA-PS (I/II vs III/IV), Mallampati class (I/II vs III/IV), procedure period (Early: July 2024–Jan 2025; Late: Feb–Jul 2025), and history of alcohol consumption or chronic use of sedative/psychotropic medications.

†Model 2 was a reduced model adjusted for key confounders only (age, ASA-PS, and history of alcohol consumption or chronic use of sedative/psychotropic medications).

^‡^Reference categories: Midazolam for sedative group, Male for sex, ASA-PS I/II, Mallampati classification I/II, Early for procedure period.
